# Stress inoculation modeled in mice

**DOI:** 10.1038/tp.2015.34

**Published:** 2015-03-31

**Authors:** J Brockhurst, C Cheleuitte-Nieves, C L Buckmaster, A F Schatzberg, D M Lyons

**Affiliations:** 1 Department of Psychiatry and Behavioral Sciences, Stanford University School of Medicine, Stanford, CA, USA

## Abstract

Stress inoculation entails intermittent exposure to mildly stressful situations that present opportunities to learn, practice and improve coping in the context of exposure psychotherapies and resiliency training. Here we investigate behavioral and hormonal aspects of stress inoculation modeled in mice. Mice randomized to stress inoculation or a control treatment condition were assessed for corticosterone stress hormone responses and behavior during open-field, object-exploration and tail-suspension tests. Stress inoculation training sessions that acutely increased plasma levels of corticosterone diminished subsequent immobility as a measure of behavioral despair on tail-suspension tests. Stress inoculation also decreased subsequent freezing in the open field despite comparable levels of thigmotaxis in mice from both treatment conditions. Stress inoculation subsequently decreased novel-object exploration latencies and reduced corticosterone responses to repeated restraint. These results demonstrate that stress inoculation acutely stimulates glucocorticoid signaling and then enhances subsequent indications of active coping behavior in mice. Unlike mouse models that screen for the absence of vulnerability to stress or presence of traits that occur in resilient individuals, stress inoculation training reflects an experience-dependent learning-like process that resembles interventions designed to build resilience in humans. Mouse models of stress inoculation may provide novel insights for new preventive strategies or therapeutic treatments of human psychiatric disorders that are triggered and exacerbated by stressful life events.

## Introduction

Stress inoculation is a form of cognitive behavioral therapy that involves intermittent exposure to mildly stressful situations for people who work in conditions where performance in the face of adversity is required, for example, medical and military personnel, police, firefighters and rescue workers.^1, 2, 3^ Exposure psychotherapies likewise train patients to imagine a graded series of stress-inducing situations and encourage interaction with stressors *in vivo*.^4^ These procedures promote learning^5^ and provide opportunities to practice acquired coping skills.^6^

Stress inoculation training sessions and exposure psychotherapies are generally administered by psychologists and psychiatrists, but these interventions build on conditions that appear to spontaneously occur without instruction or guidance.^7, 8, 9^ Mild stress exposure in childhood has been linked to lower subsequent levels of state anxiety^10^ and smaller increases in salivary cortisol responses to laboratory-based psychological stressors.^11, 12^ Prior mildly stressful experiences diminish emotional distress in workplace conditions^13^ and decrease cardiovascular responses to stressful laboratory tests.^14^ These results indicate that mild but not minimal nor severe stress exposure promotes subsequent coping and emotion regulation as described by U-shaped functions.^15, 16, 17^

Previously, we showed that stress inoculation training sessions modeled by brief intermittent social separations acutely increase cortisol and enhance subsequent indications of resilience in juvenile monkeys.^9, 18^ More recently, we found that stress inoculation is not restricted to critical or sensitive periods in development and protects adult monkeys against subsequent stress-induced anhedonia measured by sucrose preference tests.^19^ On the basis of these findings and the availability of tools for dissecting causal mechanisms that mediate experience-dependent links between behavior and brain, here we turn our attention from studies of monkeys to mice. Specifically, we test the hypothesis that stress inoculation training acutely stimulates glucocorticoid signaling and then enhances subsequent indications of resilience in mice.

## Materials and methods

C57BL/6 male mice weighing ~25 g (range 22–28 g) were purchased from Charles River (Hollister, CA, USA) and maintained in groups of two to three per cage in climate-controlled rooms with an ambient temperature of 26 °C and lights on from 0700 to 1900 h. Food and drinking water were provided *ad libitum*. After 2 weeks of acclimation, mice were randomized to stress inoculation training sessions (*n*=20) or a control treatment condition (*n*=20). For the control condition, mice remained undisturbed except for intermittent human handling during ordinary animal facility care. Age-matched mice maintained in the same conditions but randomized to stress inoculation training sessions were exposed to a standard social stress protocol developed by other investigators^20^ and modified as follows. Every other day for 21 days, mice randomized to the stress inoculation condition were removed from the home cage and individually placed for 15 min behind a mesh-screen barrier in the cage of a retired Swiss Webster male mouse breeder. Each subject was repeatedly exposed to the same resident with different residents used for different subjects to avoid idiosyncratic effects from any particular resident. The mesh-screen barrier prevented fighting and wounding during all 11 stress inoculation sessions but allowed non-contact interaction. After each session, mice were immediately returned to the home cage.

Plasma levels of the stress hormone corticosterone were assessed in undisturbed home cage baseline conditions and immediately after the first, third, seventh and eleventh stress inoculation training sessions. Corticosterone levels were also assessed after subsequent restraint stress test sessions conducted 2, 6 and 13 days following completion of the treatment conditions. Restraint stress tests consisted of confinement for 15 min every other day for seven total sessions in plastic conical 50-ml tubes perforated with ventilation holes. Tail vein blood samples were collected as described elsewhere (http://www.nc3rs.org.uk/mouse-tail-vessel-microsampling-non-surgical) between 0900 and 1030 h to control for circadian variation. Plasma extracted from blood samples was assayed in duplicate for corticosterone with a radioimmunoassay from MP Biomedicals (Santa Ana, CA, USA) without knowledge of the treatment conditions. Assay sensitivity was 7 ng ml^−1^ and the intra- and inter-assay coefficients of variation were 2.8 and 4.5%.

Blood samples for hormone measures were collected from 16 mice with *n*=8 in each treatment condition. Behavioral tests were conducted using 24 different mice with *n*=12 in each treatment condition to control for potential blood sampling effects. Sample sizes were powered to detect mean differences 80% greater than pooled variances with type I error risk of 5% and type II error probability equivalent to 80% power. Mean and variance estimates for statistical power calculations were taken from earlier monkey research.^7, 9, 18, 19^

Tail suspension tests of behavior were counterbalanced with open-field and object-exploration tests to control for test order. All behavioral tests occurred 2–13 days after completion of the treatment conditions between 0900 and 1000 h. The inter-test interval between tail-suspension and open-field tests was 5–7 days, and object-exploration tests were conducted 1 and 2 days after acclimation to the open field. Videotape records were scored by a trained observer using Noldus (Wageningen, The Netherlands) Observer XT without knowledge of the treatment conditions.

Tail suspension tests followed a protocol described by Cryan *et al.*^21^ Total time spent immobile during the 6-min test was analyzed as a standard measure of behavioral despair. Open-field tests were conducted on two consecutive days following modifications of a protocol by Gould *et al.*^22^ The mice were individually placed in a white plastic open-field box (40 × 40 × 42 cm) for each daily 10-min session. The box was cleaned after every test session. Time spent freezing was scored as the absence of all movement except respiration and is considered a measure of anxiety-like behavior in mice.^23^ Time spent within 10 cm of the walls of the box was analyzed for thigmotaxis as an additional measure of anxiety-like behavior.^24^ After acclimation to the open-field, object-exploration tests were conducted in the same box with a familiar white plastic cap from the home cage and a novel black plastic pipe. Objects were attached to the floor of the open field for each 10-min test session. The next day, exploration tests were repeated with the location of objects reversed to control for place preferences. The open-field box and objects were cleaned after each test session. Time spent exploring and latencies to explore either object were scored when an animal's head was within 1 cm from the familiar or novel object.

Data were analyzed with mixed factor analyses of variance in SYSTAT. Treatment condition was considered a between-subjects factor with test day, hormone sample condition, location within the open field and object type (novel versus familiar) considered within-subjects repeated measures. Test order was included as a statistical covariate for analyses of the behavioral measures. Relationships between measures were assessed with Pearson correlation coefficients, and all test statistics were evaluated with two-tailed probabilities (*P*<0.05).

## Results

**Figure 1:**
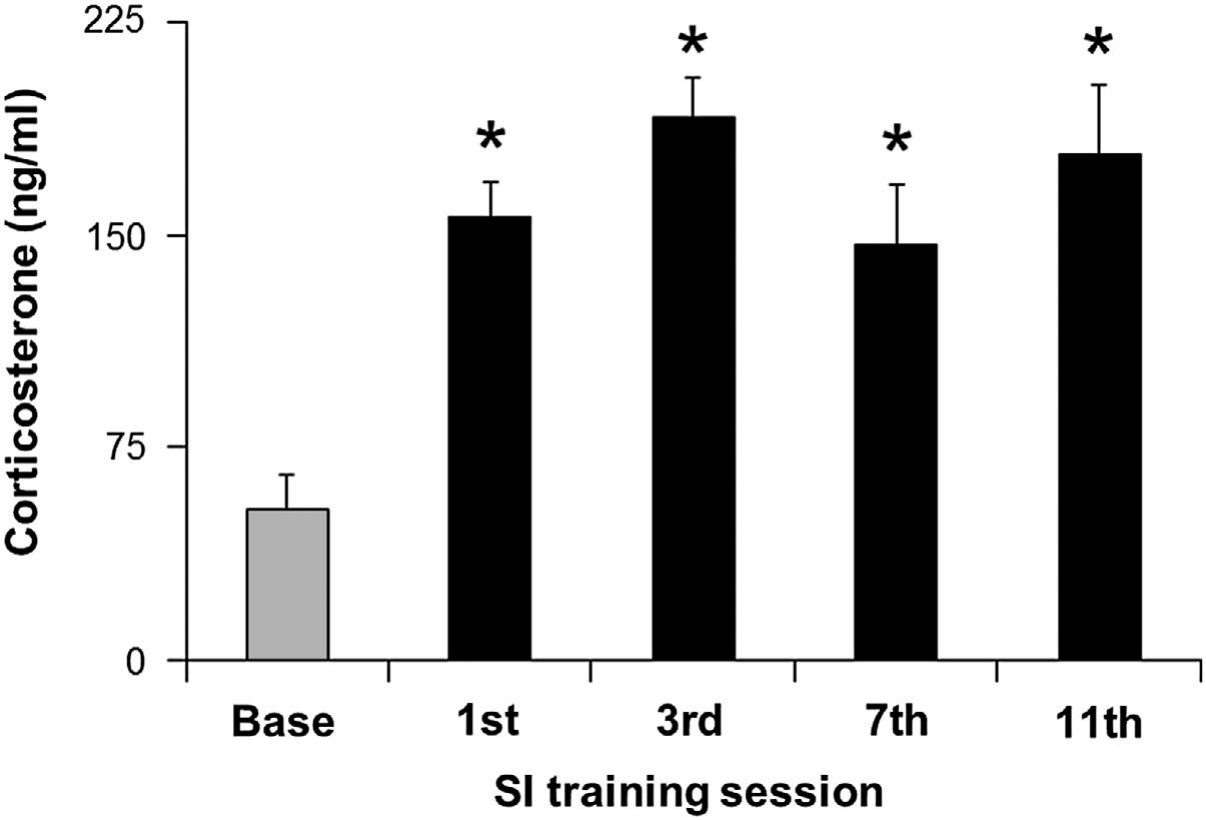
Repeated tail vein plasma corticosterone levels in undisturbed home cage baseline conditions (base) and immediately after the first, third, seventh and eleventh stress inoculation (SI) training session (mean±s.e.m., *n*=8, ^*^P<0.01, Fisher's protected *t*-tests relative to base following a repeated measures analysis of variance described in the text).

**Figure 2:**
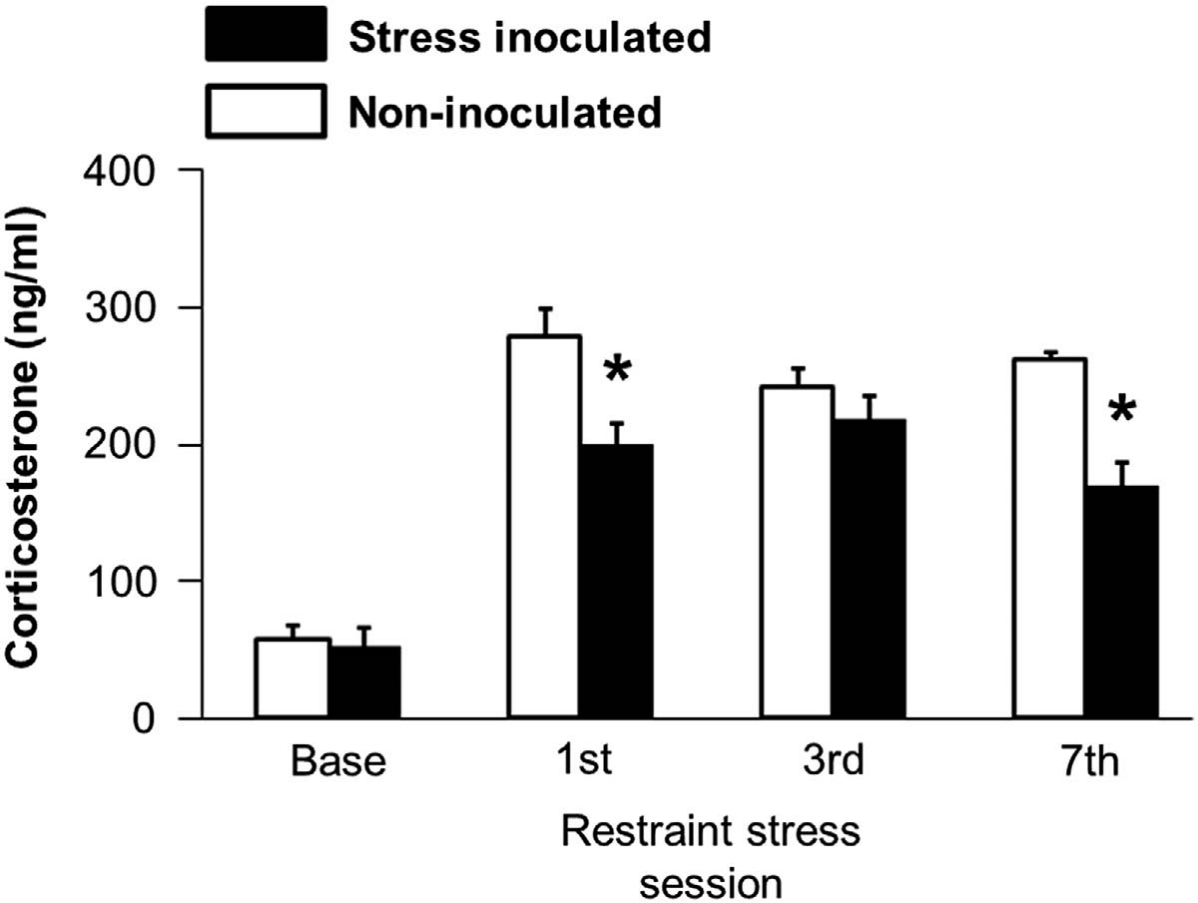
Repeated tail vein plasma corticosterone levels in undisturbed home cage baseline conditions (base) and immediately after the first, third and seventh repeated restraint stress session (mean±s.e.m., *n*=8, ^*^P<0.01, Fisher's protected *t*-tests following a treatment-by-sample condition interaction described in the text).

Stress inoculation training sessions consistently elicited robust corticosterone responses (Figure 1) as discerned by analysis of variance (F(4,28)=13.19, *P*<0.001). Stress inoculation subsequently diminished corticosterone responses to restraint (Figure 2) as indicated by a treatment main effect (F(1,14)=15.57, *P*=0.001), sample condition main effect (F(3,42)=91.33, *P*<0.001) and treatment-by-sample condition interaction (F(3,42)=5.33, *P*=0.003). During restraint, we informally noted that struggling behavior appeared to occur more often in stress-inoculated mice compared with controls. We tested this hypothesis in a different sample of mice using tail-suspension tests.

**Figure 3:**
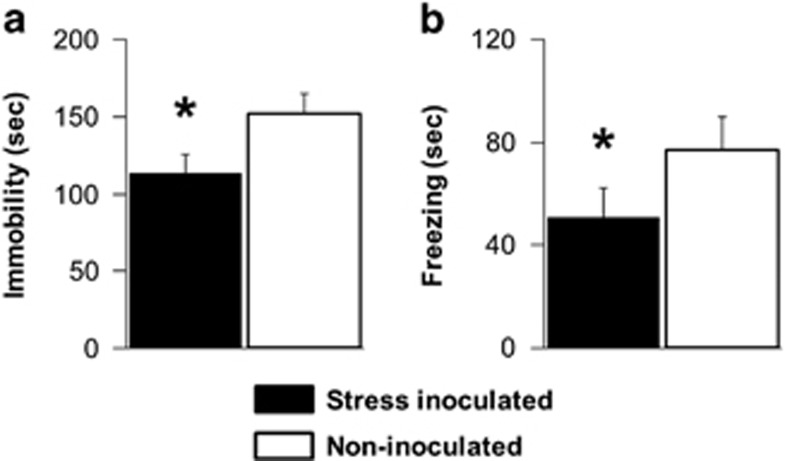
Stress inoculation training subsequently decreased (**a**) immobility on tail-suspension tests and (**b**) freezing in the open field (mean±s.e.m., *n*=12, ^*^P<0.05, Fisher's protected *t*-tests following analyses of variance described in the text). Note: different y axis time scales for each graph.

Stress-inoculated mice spent significantly less time immobile as a measure of behavioral despair on tail-suspension tests compared with controls (F(1,21)=6.38, *P*=0.021; Figure 3a). Anxiety-like behavior indexed by mean freezing scores from two open-field tests was also diminished by prior stress inoculation compared with controls (F(1,21)=5.98, *P*=0.023; Figure 3b). Open-field test results for freezing were consistent over repeated days (data not shown) as the test day main effect (*P*=0.104) and test day-by-treatment interaction (*P*=0.765) were not significant. Significant treatment differences in freezing were evident despite evidence that open-field tests evoked thigmotaxis as a measure of anxiety-like behavior in both treatment conditions (data not shown). Time spent close to the walls was nearly eightfold greater than time spent in the center of the open field, and the treatment main effect (*P*=0.760) and treatment-by-test day interaction (*P*=0.455) were not significant for the measure of thigmotaxis.

**Figure 4:**
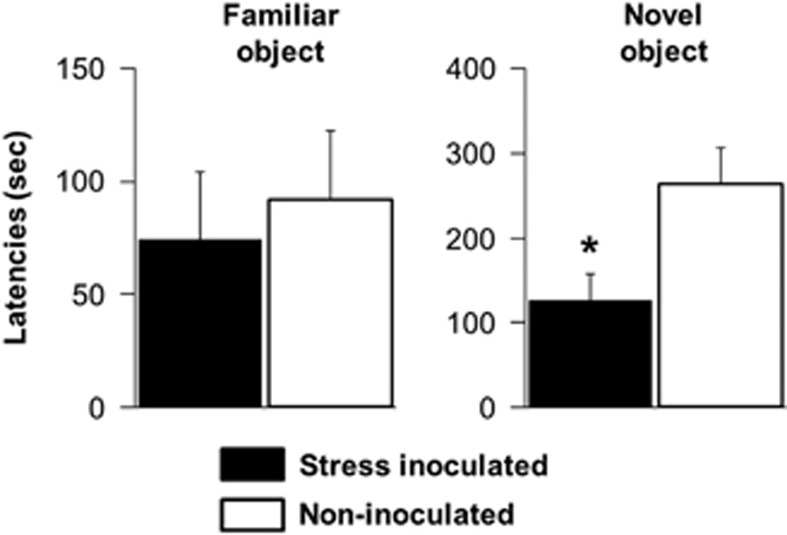
Stress inoculation training subsequently decreased novel-object exploration latencies (mean±s.e.m., *n*=12, ^*^P=0.017, Fisher's protected *t*-test following analyses of variance described in the text). Note: different y axis time scales for each graph.

During object-exploration tests, object type and treatment main effects were discerned for latency scores as depicted in Figure 4. Shorter latencies were evident for exploration of the familiar compared with novel object (F(1,21)=9.02, *P*=0.007) and stress-inoculated mice explored objects faster than non-inoculated controls (F(1,21)=6.06, *P*=0.023). The object type-by-treatment interaction was not significant (*P*=0.121), but stress-inoculated mice explored the novel object faster than non-inoculated controls (F(1,21)=6.76, *P*=0.017). Treatment differences were not significant (*P*=0.695) for latencies to explore the familiar object (Figure 4). Shorter exploration latencies were noted on the first compared with the second test day (F(1,21)=5.06, *P*=0.035; data not shown) but the test day-by-treatment (*P*=0.124) and test day-by-treatment-by-object type interactions (*P*=0.308) were not significant.

Time spent exploring the novel object was, on average, 22% greater in stress-inoculated mice compared with non-inoculated controls (data not shown) but the treatment main effect (*P*=0.452) and treatment-by-test day interaction (*P*=0.429) were not significant. Mice from both treatment conditions that spent more time exploring the novel object across repeated test days tended to show shorter novel-object exploration latencies, but the correlation was not quite significant (*r*=−0.34, df 22, *P*=0.10).

## Discussion

Stress inoculation training sessions acutely increased plasma levels of corticosterone in mice and then diminished subsequent immobility on tail-suspension tests. Stress inoculation also decreased subsequent freezing in the open field despite comparable levels of thigmotaxis as a measure of anxiety-like behavior in mice from both treatment conditions. Stress inoculation subsequently decreased latencies for novel-object exploration consistent with earlier studies of monkeys,^25^ and reduced corticosterone responses to repeated restraint. These results demonstrate that stress inoculation training acutely stimulates glucocorticoid signaling and then enhances subsequent indications of active coping behavior in mice.

Stress inoculation training sessions for mice were designed on the basis of evidence that mild but not minimal nor severe stress exposure provides opportunities to learn, practice and improve coping as described by U-shaped functions.^15, 16, 17^ We modified a standard social stress protocol^20^ to generate a mildly stressful experience without fighting, wounding or direct forms of contact aggression. Instead of daily exposure and continuously living in the presence of an aggressive resident, stress inoculation training sessions were conducted every other day with subjects returned to the home cage immediately after completion of each training session. These modifications allowed ample time for recovery and consolidation of memory in undisturbed home cage conditions. Studies of human handling, transportation and other routine procedures in rodent research facilities are needed to delineate specific conditions for producing mildly stressful experiences with inoculation effects in mice.

Mildly stressful experiences are a key feature of stress inoculation training for humans^1, 2, 3^ and monkeys.^9, 18, 19^ Primate models are important because the behavior and neurobiology of monkeys more closely resemble humans than do models based exclusively on rodents.^26, 27^ Nevertheless, the availability of tools for dissecting causal mechanisms that mediate experience-dependent links between behavior and brain is far greater in mice than monkeys.^28, 29^ In this regard, mouse models offer uniquely important translational opportunities to bridge the gap between basic and clinical psychiatry research.

Future studies of mice may provide novel insights on neuroplasticity and stress inoculation-induced aspects of behavior change. Stress inoculation training in monkeys enhances adult hippocampal neurogenesis and alters the expression of genes involved in cell proliferation and survival.^7^ Antidepressant medications likewise increase hippocampal neurogenesis in humans^30^ and decrease immobility on tail-suspension tests in mice.^21^ Optogenetic manipulations of adult hippocampal neurogenesis^31, 32^ may uncover causal connections between this aspect of neuroplasticity and active coping behavior induced by stress inoculation in mice.

Glucocorticoid signaling during stress inoculation training suggests additional opportunities for translational research on the basis of evidence that glucocorticoid administration enhances the efficacy of exposure psychotherapies for human anxiety disorders.^33^ Stress inoculation also acutely elevates endogenous glucocorticoid (that is, corticosterone) levels without habituation in mice. Although repeated exposure to homotypic stressors generally elicits habituation of the corticosterone response,^34^ repeated non-contact exposure to fighting between same-sex conspecifics does not result in habituation over 10 successive sessions.^35^ Moreover, we found no published evidence that brief non-contact exposure to an individual social stranger increases corticosterone without habituation as observed here in mice. Previously, we reported that stress inoculation enhances glucocorticoid receptor expression in monkey anterior cingulate cortex but not neurogenic regions of adult hippocampus.^19^ Glucocorticoid receptors are ligand-activated transcription factors that translate circulating glucocorticoid levels into genomic outputs by binding DNA and regulating the expression of numerous genes involved in neuroplasticity and behavior change.^36, 37, 38, 39^ Genetically engineered mice with altered glucocorticoid receptors in anterior cingulate cortex may help to identify causal roles for glucocorticoid signaling in stress inoculation and related exposure psychotherapies.

These suggestions reflect a new strategy for translational psychiatry. In addition to investigating how the effects of severe stress damage behavior and brain,^40, 41, 42^ we propose a complementary approach focused on stress inoculation. Mechanisms that mediate stress inoculation in animals may provide novel targets for the development of new preventive or therapeutic interventions for humans. Pharmacological mimicry of stress inoculation is a novel approach that shifts attention from neuropathology to consider the mechanisms that mediate experience-dependent coping as new targets for interventions.

Our results should be interpreted in the context of potential limitations. Findings from males may or may not hold true for females. Sex differences in emotionality and stress hormone responses have been reported for rats^43, 44^ and sex differences may warrant attention in mice. Stress inoculation training sessions and subsequent test procedures both required transfer of mice into new environments. Studies of habituation or extinction of fear from repeated transfers alone are needed but the contextual differences between training and our test procedures generally increase emotional responses^34, 45^ instead of producing the observed stress inoculation effects. Correlations between hormones and behavior are not provided because these measures were collected from different animals to control for potential blood sampling effects. Moreover, the statistical power to detect correlations between behavioral measures was limited by standard sample sizes used in this research.

In summary, we found that stress inoculation training sessions acutely increase plasma levels of corticosterone and then protect against subsequent immobility on tail-suspension tests. Stress inoculation also decreases subsequent freezing in the open field, decreases latencies for novel-object exploration and reduces corticosterone responses to repeated restraint. Unlike mouse models that screen for the absence of vulnerability to stress or presence of traits that occur in resilient individuals,^17, 46^ stress inoculation training in mice is an experience-dependent learning-like process that resembles interventions designed to build resilience in humans. Mouse models of stress inoculation may provide novel insights for new preventive strategies or therapeutic treatments of human disorders that are triggered and exacerbated by stressful life events.

## Notes

AFS reports equity in Merck, Pfizer, Neurocrine, XHale and Corcept Therapeutics (co-founder). AFS has received lecture fees from Merck and consulted to Takeda/Lundbeck, Pfizer, Depomed and Neuronetics. The remaining authors declare no conflict of interest. All the authors are employed by or were students at Stanford University.
